# C-C Chemokine Ligand-5 is critical for facilitating macrophage infiltration in the early phase of liver ischemia/reperfusion injury

**DOI:** 10.1038/s41598-017-03956-7

**Published:** 2017-06-16

**Authors:** Chiou-Mei Lee, Hsin-Hsin Peng, Polung Yang, Jiin-Tarng Liou, Chia-Chih Liao, Yuan-Ji Day

**Affiliations:** 1 Laboratory Animal Center, Chang Gung Memorial Hospital, Linkou, Taiwan; 2 Center for Molecular and Clinical Immunology, Chang Gung University, Chang Gung, Taiwan; 3 Department of Anesthesiology, Chang Gung Memorial Hospital, Linkou, Taiwan; 4 Molecular Medicine Research Center, Chang Gung University, Chang Gung, Taiwan; 5 Department of Anesthesiology, Hualien Tzu Chi Hospital, Tzu Chi University, Buddhist Tzu Chi Medical Foundation, Hualien, Taiwan

## Abstract

CCL5/RANTES, a chemoattractant for myeloid cells, is induced by hepatic ischemia/reperfusion injury (IRI). The roles of CCL5 in hepatic IRI were carried out by means of CCL5 immunodepletion, antagonistic competition by Met-CCL5, and treatment with recombinant murine CCL5 (rmCCL5). Depletion or inhibition of CCL5 reduced severity of hepatic IRI, whereas rmCCL5 treatment aggravated liver IRI as manifested in elevated serum alanine aminotransferase (ALT) and tissue myeloperoxidase (MPO) levels. Moreover, IRI severity was reduced in CCL5-knockout (CCL5-KO) mice versus wildtype (WT) mice, with drops in serum ALT level, intrahepatic MPO activity, and histological pathology. Bone marrow transplantion (BMT) studies show that myeloid cells and tissue cells are both required for CCL5-aggravated hepatic IRI. The profile of liver-infiltrating leukocyte subsets after hepatic reperfusion identified CD11b+ cells as the only compartment significantly reduced in CCL5-KO mice versus WT controls at early reperfusion phase. The role of CCL5 recruiting CD11b+ cells in early reperfusion was validated by *in vitro* transwell migration assay of murine primary macrophages (broadly characterized by their CD11b expression) in response to liver lysates after early reperfusion. Taken together, our results demonstrate a sequence of early events elicited by CCL5 chemoattracting macrophage that result in inflammatory aggravation of hepatic IRI.

## Introduction

Ischemia/Reperfusion injury (IRI) is a major concern of surgical procedures involving liver, especially major hepatic resection and liver organ transplantation, which subject hepatic tissues to ischemic conditions^1^. During reperfusion, blood flow is restored to the ischemia-injured tissues. Subsequent respiratory burst results in generation of reactive oxygen species (ROS), and cellular destruction by free radicals and subsequent initiation of inflammatory cascades lead to activation and further recruitment of leukocytes, which aggravate the extent of inflammation and injury^2, 3^.

CCL5 is a β-chemokine that is the ligand for chemokine receptors CCR1, CCR3 and CCR5. CCL5 mediates recruitment and migration of T lymphocytes and macrophages^4, 5^ and is expressed by a wide variety of tissues including epithelial cells, fibroblasts, and platelets^4^. At high concentration, multimeric CCL5 is capable of sustaining leukocyte activation and the ensuing inflammatory responses^4^. In addition to liver ischemia reperfusion, CCL5 expression is also found to be elevated in ischemia-reperfusion injuries of brain, lung, heart, kidney, intestine, and skeletal muscle^6–12^. In atherosclerotic mice, neutralization of CCL5 signaling by CCR5 antagonist ameliorated myocardial reperfusion injury^6^. CCL5 also mediates inflammation and tissue injury in a model of induced focal ischemia-reperfusion in the murine brain^13^. The cerebral ischemia-reperfusion model in particular implicated that CCL5 derived from myeloid cells might at least partially contribute to reperfusion-induced inflammation, in addition to the chemotactic signal released by the injured tissues.

While the roles of CCL5 in mediating reperfusion-related injury of cerebellum and cardiac muscle have been documented^6, 13^, the extent and mechanism of its actions in the context of liver ischemia-reperfusion-induced inflammation remain to be elucidated. In this study, we report that CCL5 contributes to the pathology of hepatic injuries caused by ischemia reperfusion. The extent of CCL5 involvement in IRI was demonstrated by CCL5-targeting treatments that led to alleviated IRI-induced liver tissue damage. These treatments include gene depletion or functional disruption of CCL5 by various approaches including IR-induction in CCL5-knockout mice, intravenous (i.v.) CCL5 neutralization by CCL5-neutralizing antibody, and i.v. competition for CCL5 receptors by Met-CCL5, a CCL5 chemokine receptor antagonist that blocks the effects of CCL5. In contrast, i.v. recombinant CCL5 treatment of wt mice immediately at reperfusion resulted in aggravated severity of liver IRI. Furthermore, we have shown with BMT mice models that myeloid cells and tissue-derived CCL5 are both essential contribution in liver IR injury, and defective CCL5 expression resulted in impairment of leukocyte infiltration. In liver ischemic stroke, macrophages infiltrate early and accumulate in localized lesions, contributing to aggravation of inflammatory damages of IRI-afflicted tissues. It is notable that mice with CCL5 deficiency exhibited significantly reduced macrophage recruitment during early reperfusion following 1 hr liver ischemia. These results show that CCL5 facilitates macrophage recruitment to the site of liver IRI, leading to pro-inflammatory inductions and subsequent injuries. Thus CCL5 activity may represent a potential target for novel therapeutic strategies against ischemia-reperfusion damages during hepatic operations.

## Results

### Immunodepletion of CCL5 alleviated hepatic ischemia-reperfusion injury (IRI)

**Figure 1:**
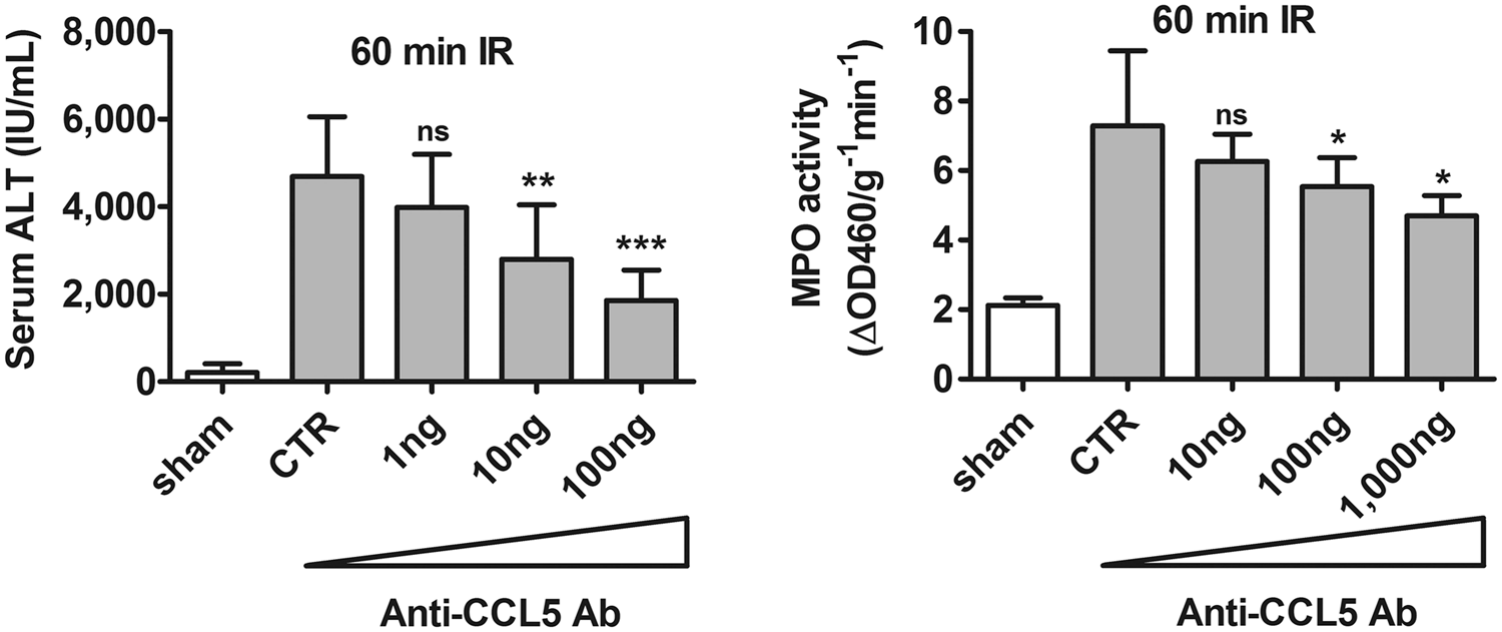
Immunodepletion of CCL5 alleviated inflammatory hepatic IRI. C57BL/6C mice were subjected to ischemia for 60 minutes and received anti-CCL5 antibody via jugular vein immediately after reperfusion. Mice were sacrificed at 24 hours after reperfusion. Mice administered with either IgG control or anti-CCL5 Abs (1, 10, and 100 ng) for immunodepletion of CCL5. Both the serum ALT and tissue MPO activities decreased significantly in a dose dependent manner (Mean ± SD, n = 8). CTR, control. ns = not significant at *p* > 0.05; *p < 0.05; **p < 0.01, ***p < 0.0001.

In our previous studies, we have shown that ATL146e, an agonist of A_2A_AR, alleviated hepatic IRI in conjunction with reduction of the expression levels of proinflammatory cytokines, in particular CCL5^14–16^. These data implicate CCL5 as a critical mediator of inflammatory hepatic IR injury. In order to elucidate the roles of CCL5 in hepatic IRI, we manipulated the intrahepatic level of CCL5 in a murine model of partial warm hepatic IRI by administration of CCL5-specific agents through jugular vein injection immediately after reperfusion of the liver at the end of ischemia that lasted 60 minutes. These agents included CCL5-specific neutralizing antibodies (Ab), which clear endogenous CCL5 by immunodepletion; Met-CCL5, a CCL5 chemokine receptor antagonist (CCR1 and CCR5); and recombinant murine CCL5 (rmCCL5). In the immunodepletion study, the CCL5-neutralizing antibodies at progressively higher doses inhibited IR injury in a dose-dependent manner, as assessed by serum ALT and parenchyma tissue MPO activities (Fig. 1). Serum ALT levels following IRI were 3,894 ± 1,211, 2,794 ± 1,253 and 1,857 ± 692 (IU/mL) in wt mice with treatments of 1, 10, and 100 ng of anti-CCL5 antibodies respectively (*p* = 0.28, <0.001, and <0.0001, respectively, versus the IgG control mice (“CTR” in Fig. 1 (left panel); 4,689 ± 1,366). At the maximum injection dose of anti-CCL5 antibodies tested (100 ng), the post-IRI serum ALT levels of the recipient mice dropped to approximately 40% of the level observed in control animals receiving non-specific isotype IgG (left panel in Fig. 1). Similar trend was observed for intrahepatic MPO activity in wt mice given increasing anti-CCL5 antibody doses of 10, 100, and 1,000 ng, with the corresponding values being 6.3 ± 0.8, 5.5 ± 0.8, and 4.7 ± 0.6 (ΔOD460/g^−1^ min^−1^), respectively (*p* = 0.23, *p* < 0.05, and 0.01, respectively, versus the MPO value of 7.3 ± 2.2 in IgG treated control mice (“CTR”); right panel in Fig. 1). These results represent a significant reduction of post-IRI intrahepatic MPO activity in anti-CCL5 Ab-treated mice reaching ~64% of the control levels at the maximum dose tested (right panel in Fig. 1).

### Inhibition of hepatic IRI by CCL5 receptor antagonist

**Figure 2:**
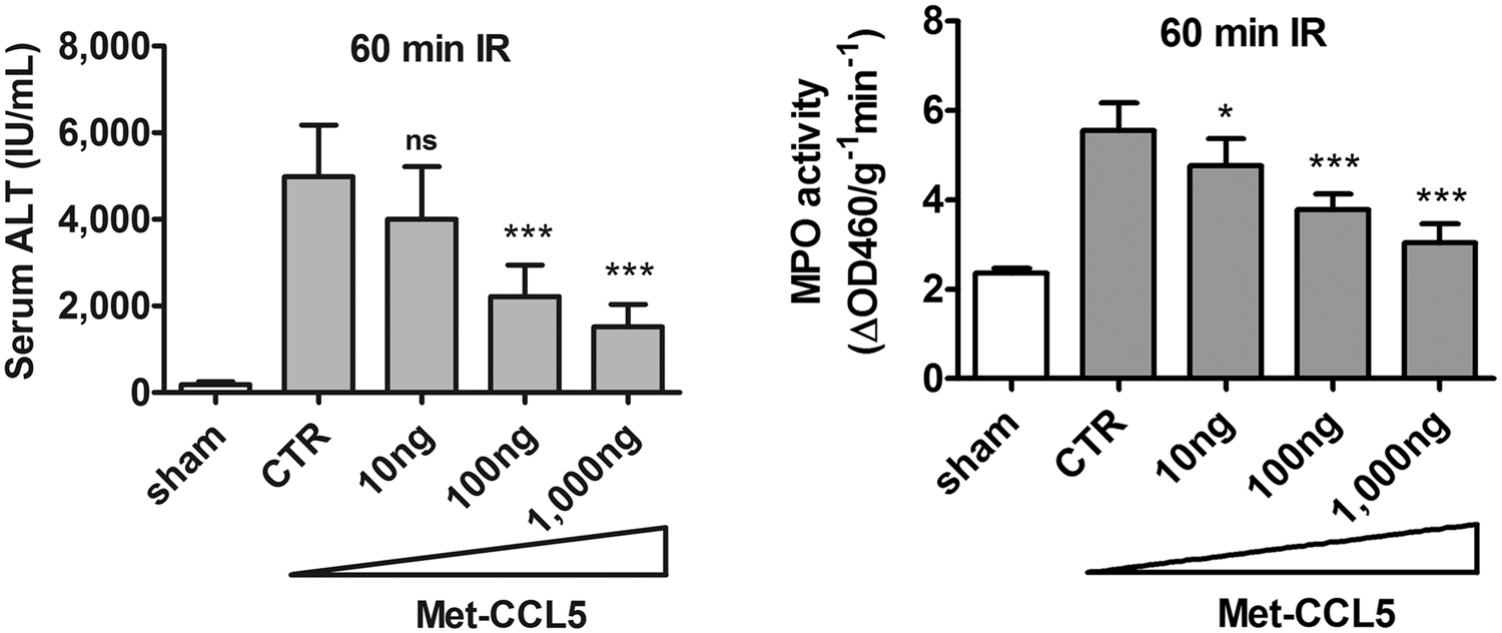
Inhibition of inflammatory hepatic IR injury by Met-CCL5. C57BL/6C mice were subjected to ischemia for 60 minutes and received escalating doses of Met-CCL5 (0, 10, 100 and 1,000 ng) via jugular vein immediately after reperfusion. Mice were sacrificed at 24 hours after reperfusion. Both the serum ALT and tissue MPO activities decreased significantly in a dose dependent manner (Mean ± SD, n = 10, ischemic time = 60 min). CTR, control. ns = not significant at *p* > 0.05; *p < 0.05; **p < 0.01, ***p < 0.0001.

An *in vivo* competition against CCL5 for receptor binding was further performed using Met-CCL5, an antagonist of CCR1 and CCR5 receptor. Treatment with escalating doses of Met-CCL5 at 10, 100, and 1,000 ng per animal immediately after reperfusion at the end of ischemia that lasted 60 minutes in wt mice successfully alleviated the severity of hepatic IRI in a dose-proportionate manner (Fig. 2). The serum ALT level following IRI was 4,005 ± 1,211, 2,216 ± 729, and 1,518 ± 516 (IU/mL) for wt mice with 10, 100, and 1,000 ng of Met-CCL5 treatment, respectively (*p* = 0.11, <0.0001, and <0.0001, respectively), versus the value of 4,689 ± 1,366 in serum ALT level of vehicle treated control mice (‘CTR’, left panel in Fig. 2). Similarly, the intrahepatic MPO activity was 4.8 ± 0.6, 3.8 ± 0.3, and 3.0 ± 0.4 ΔOD460/g^−1^ min^−1^ in wt mice administered with the same escalating doses of Met-CCL5 at 10, 100, and 1,000 ng per animal, respectively (*p* < 0.05, 0.0001, and 0.0001 respectively vs. vehicle control; right panel in Fig. 2). Again, the assessment of serum ALT level and intrahepatic MPO activity, respectively, showed that at the maximum dose, Met-CCL5 suppressed the ALT level to approximately 30% of the vehicle control treatment, while at the same time intrahepatic MPO activity dropped to about 55% of control (Fig. 2). These results were consistent with the observations made with CCL5-immunodepleted mice undergoing liver IRI.

### Induction of hepatic ischemia-reperfusion injury by rmCCL5

**Figure 3:**
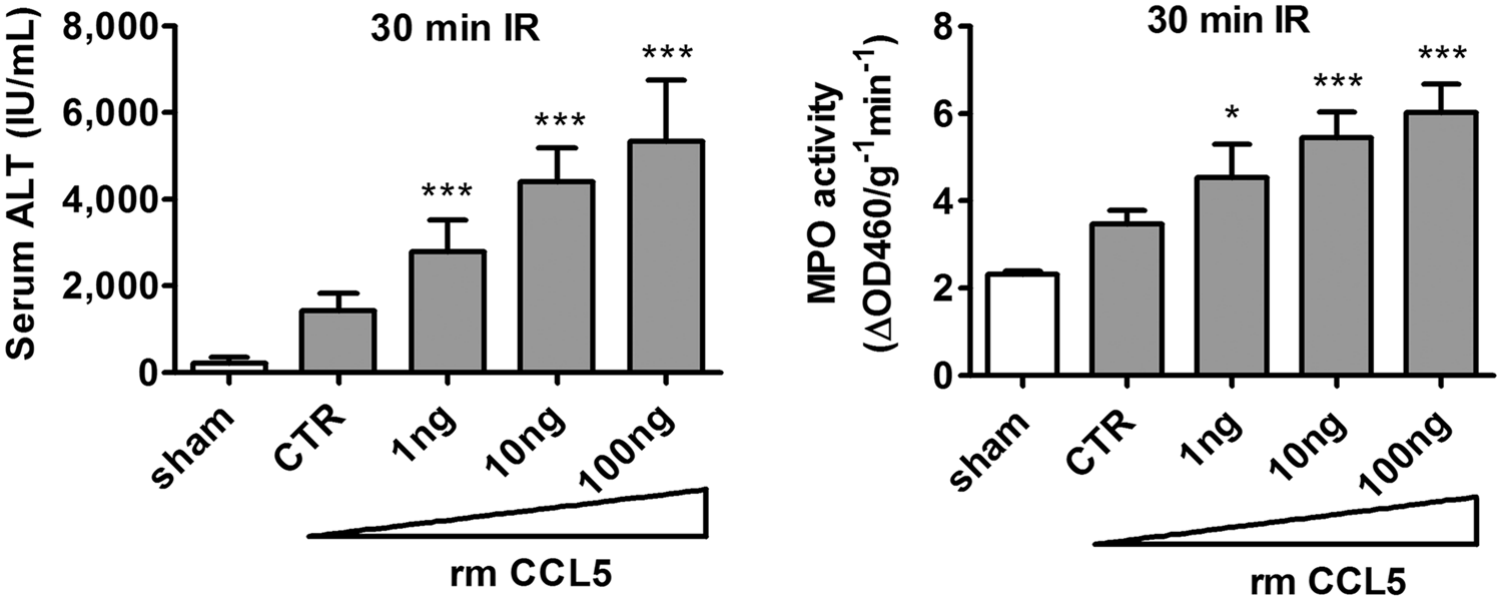
CCL5 introduction aggravated inflammatory hepatic IRI. C57BL/6C mice were subjected to 30 minutes of ischemia to allow for observation of aggravation of inflammatory injury in viable animals. Mice were dosed with vehicle control buffer or rmCCL5 in escalating doses (0, 1, 10 and 100 ng per animal) via jugular vein immediately after reperfusion. Mice were sacrificed at 24 hours after reperfusion. The serum ALT and tissue MPO activities increased in a dose-dependent manner (Mean ± SD, n = 8). CTR, control. *p < 0.05; ***p < 0.0001.

To confirm the functional role of CCL5 in facilitating hepatic ischemia-reperfusion injury, we injected rmCCL5 into liver post ischemia-reperfusion to test its ability to aggravate reperfusion injuries. Because post ischemia-reperfusion treatment with rmCCL5 after 60 minutes of ischemia resulted in mortality of the treated animals, the length of ischemia was shortened to 30 minutes in order to elucidate the extent of hepatic IRI aggravation by rmCCL5 in viable animals. The serum ALT levels of mice treated with increasing doses of 1, 10, and 100 ng of rmCCL5 during IR reached 2,788 ± 741, 4,406 ± 784, and 5,353 ± 1,397 (IU/mL), respectively (*p* < 0.0001 for all results versus the control (‘CTR’) value of 1,424 ± 405 IU/mL, left panel in Fig. 3). Similarly, the post-IRI intrahepatic MPO activity was 4.53 ± 0.8, 5.4 ± 0.6, and 6.0 ± 0.7 ΔOD460/g^−1^ min^−1^ for treatments with 1, 10, and 100 ng of rmCCL5 respectively (*p* < 0.05, 0.0001, and 0.0001, respectively, vs. control value of 3.5 ± 0.3 ΔOD460/g^−1^ min^−1^ in CTR samples; right panel in Fig. 3). In short, rmCCL5 aggravates the severity of hepatic IRI in a dose-proportionate manner, as exemplified in both serum ALT level and intrahepatic MPO activity that elevated with each escalating dose of rmCCL5. The dose-proportionate IR aggravation by rmCCL5 treatment, when contrasted with the dose-proportionate IR-alleviation by CCL5-depletion or CCL5 receptor antagonism, confirmed the dose-dependent, stimulatory effects of CCL5 during liver IR and further demonstrated the essential role of CCL5 in inflammatory hepatic IR injuries.

### Loss of CCL5/RANTES expression suppressed the extent of induced hepatic IR injury in mice

**Figure 4:**
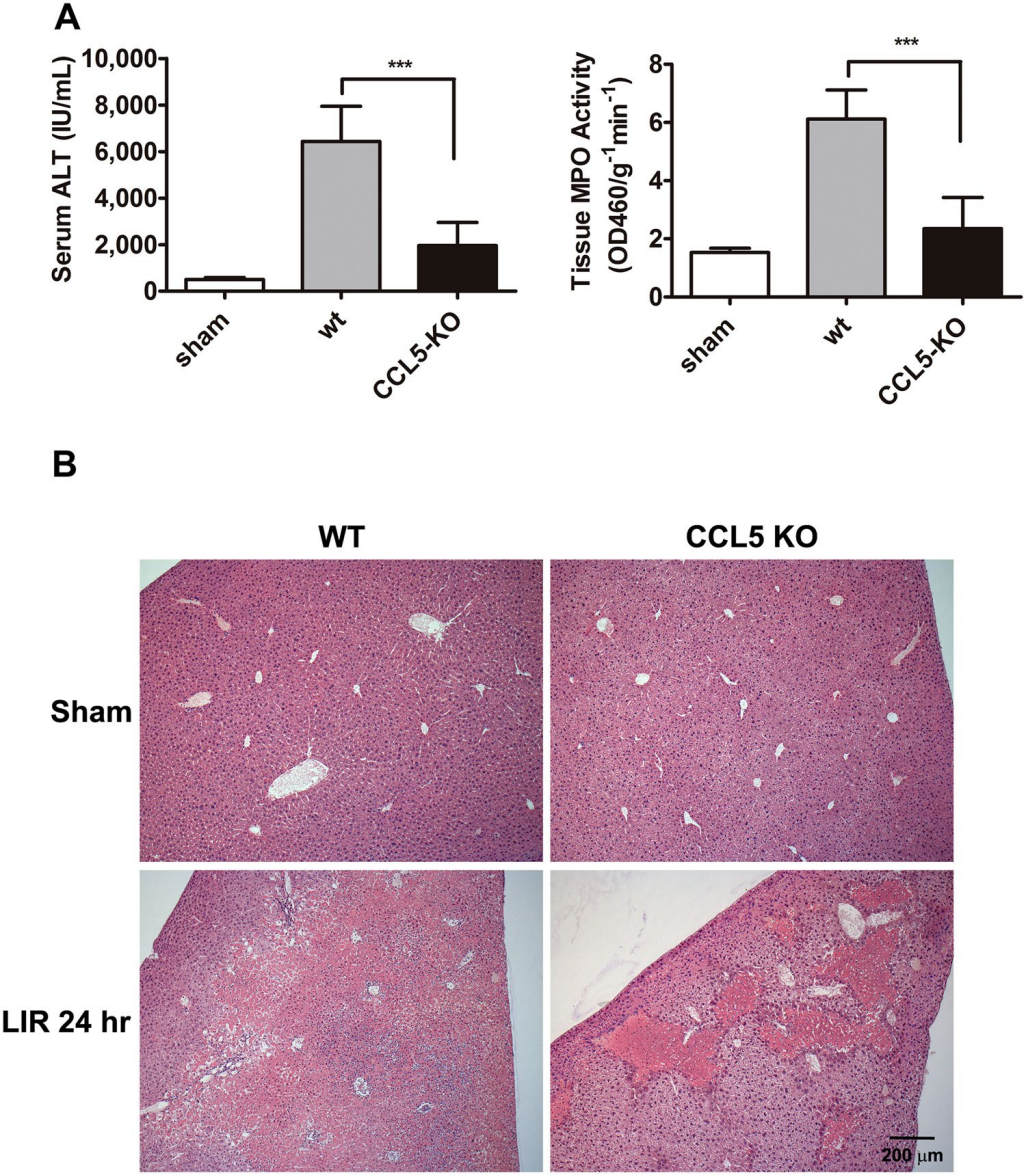
Mice with deletion of the CCL5 gene exhibited alleviated post-IR injury. Both C57BL/6 C and CCL5-KO mice were subjected to 60 min ischemia followed by 24 hours reperfusion. (**A**) The serum ALT and tissue MPO activities were measured in B6 and CCL5-KO mice (Mean ± SD, n = 12). ***p < 0.0001. (**B**) Histological studies (H&E stains) showed broad and widespread area of necrosis found in wildtype mice. A much smaller and more confined area of necrosis was observed in CCL5-KO mice. Scale bar = 200 μm.

To further validate the contribution of CCL5 to the hepatic IR injury and inflammation, we induced hepatic IRI in CCL5-KO or wt mice by clamping the medium and left hepatic triad for one hour followed by reperfusion for 24 hours. In the absence of CCL5 expression, CCL5-KO mice undergoing liver ischemia/reperfusion exhibited alleviated reperfusion injury versus wt mice, as assessed by serum ALT level and intrahepatic MPO activity (Fig. 4A). In wt mice, the level of serum ALT rose following IRI to 6,437 ± 1,510 as compared to 505 ± 278 IU/mL in sham but was reduced significantly to 1,958 ± 998 IU/ml in CCL5-KO mice (*p* < 0.0001 compared with the value of 6,437 ± 1,510 IU/ml in wt control, left panel in Fig. 4A). Observations of IR-associated change of intrahepatic MPO activity in CCL5-KO mice also revealed similar patterns (Fig. 4A, right panels). The MPO activity dropped significantly to 2.4 ± 1.1 ΔOD460/g^−1^ min^−1^ in CCL5-KO mice versus the control value of 6.1 ± 1.0 ΔOD460/g^−1^ min^−1^ in wt mice (*p* < 0.0001, right panel in Fig. 4A). Furthermore, histological examination of the hepatic IRI tissues of CCL5-KO mice by hematoxylin and eosin (H&E) stain revealed a dramatic reduction of tissue necrosis to a limited, confined region of lesion in the hepatic tissues, as opposed to widespread tissue damages seen for wt mice undergone identical treatment of induced liver IRI (Fig. 4B). Consistent with our earlier observations with exogenous manipulation of anti-CCL5 or Met-CCL5 availability (Figs 1 and 2), these results confirm that CCL5 directly contributes to the severity of hepatic IRI.

**Figure 5:**
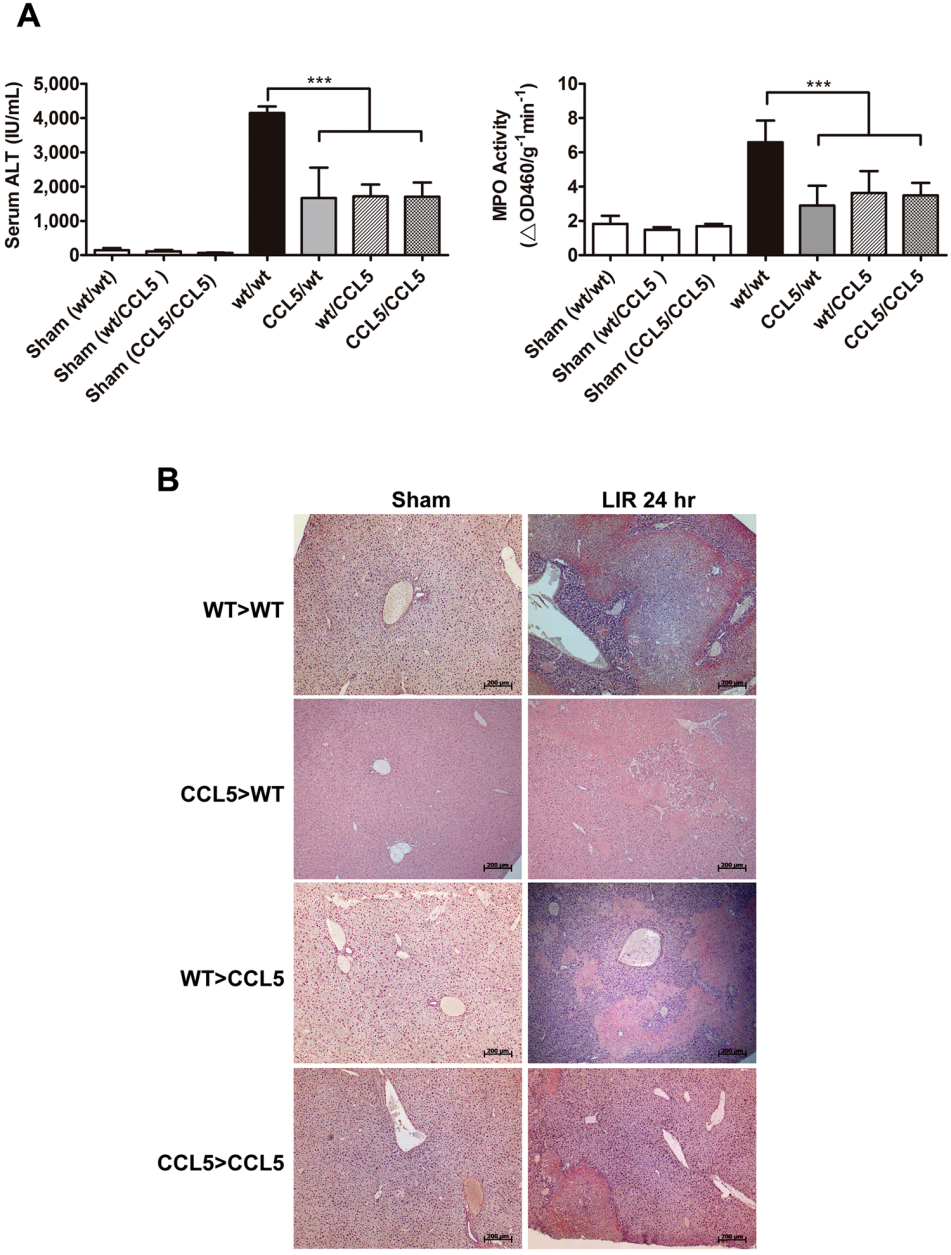
Deficiency in blood cell-and tissue-derived CCL5 resulted in reduced liver damages. C57BL/6C or CCL5-KO mice receiving either wildtype or CCL5-KO bone marrow (BM) cells after lethal irradiation were studied after 8 weeks of repopulation (BMT chimera denoted by donor > recipience). The BMT chimera mice were subjected to 60 min of ischemia and 24 hours of reperfusion. (**A**) The serum ALT and tissue MPO activities were evaluated in BMT chimera. The results demonstrated that CCL5 deficient chimera of CCL5 > WT, WT > CCL5, and CCL5 > CCL5 had significantly lower level of ALT and MPO activities compared to WT > WT chimera (Mean ± SD, n = 10). ***p < 0.0001. (**B**) The histology (H&E stains) studies showed less area of necrosis in tissue sections obtained from BMT chimera. Scale bar = 200 μm.

The direct involvement of CCL5 in the aggravation of hepatic IRI raises the question as to the exact source of endogenous CCL5 in the course of injury. It is suggested that during ischemia/reperfusion, circulating blood cells reaching the ischemia site might be as another origin of CCL5 expression in addition to tissue parenchyma, and the resultant IR injury^3, 13^. Therefore, we further evaluated the involvements of circulating peripheral blood cells and hepatic tissue cells during CCL5-induced liver IR injury in a BMT chimera model. Bone marrows from wt or CCL5-KO donor mice were used to reconstitute those of irradiated wt or CCL5-KO recipient mice. The BMT-reconstituted mice were then subject to induction of hepatic IRI. The CCL5/wt (donor/recipient) BMT mice underwent a significant reduction in liver damages versus wt/wt chimera controls, with the serum ALT level and intrahepatic MPO activity reaching 1,666 ± 888.4 IU/mL and 2.9 ± 1.2 ΔOD460/g^−1^ min^−1^, respectively, compared to their respective wt/wt controls (*p* < 0.0001 for both assays of ALT and MPO in wt/wt controls; the control ALT level was 4,143 ± 741.6 IU/mL, and the control MPO activity was 6.6 ± 1.3 ΔOD460/g^−1^ min^−1^; Fig. 5A). However, there was no significant difference in liver damages of wt/CCL5 BMT animals versus that of CCL5/CCL5 BMT animals (*p* > 0.05 for both assays of ALT and MPO in wt/CCL5 and CCL5/CCL5 mice; the respective ALT levels were 1,713 ± 1,043 IU/mL in wt/CCL5 mice and 1,703 ± 1,311 IU/mL in CCL5/CCL5 mice, and the intrahepatic MPO activity levels were 3.6 ± 1.3 ΔOD460/g^−1^ min^−1^ and 3.5 ± 0.7 ΔOD460/g^−1^ min^−1^ respectively; Fig. 5A). The observed serum ALT level and liver MPO activity in these BMT chimera mice were consistent with the results of liver histopathology study by H&E staining showing reduced hepatic tissue damages in all chimeras versus wt/wt (Fig. 5B). In other words, CCL5 gene knockout in either circulating bone marrow-derived cells or resident hepatic tissue cells was sufficient to significantly reduce the severity of IR-induced injury as indicated by both serum ALT level and intrahepatic MPO activity. Conversely, expression of CCL5 by both circulating and resident cells is necessary to facilitate post-ischemia/reperfusion hepatic inflammation and tissue damage to the fullest extent.

### CCL5 is essential for macrophage infiltration at early stage during liver IR injury

**Figure 6:**
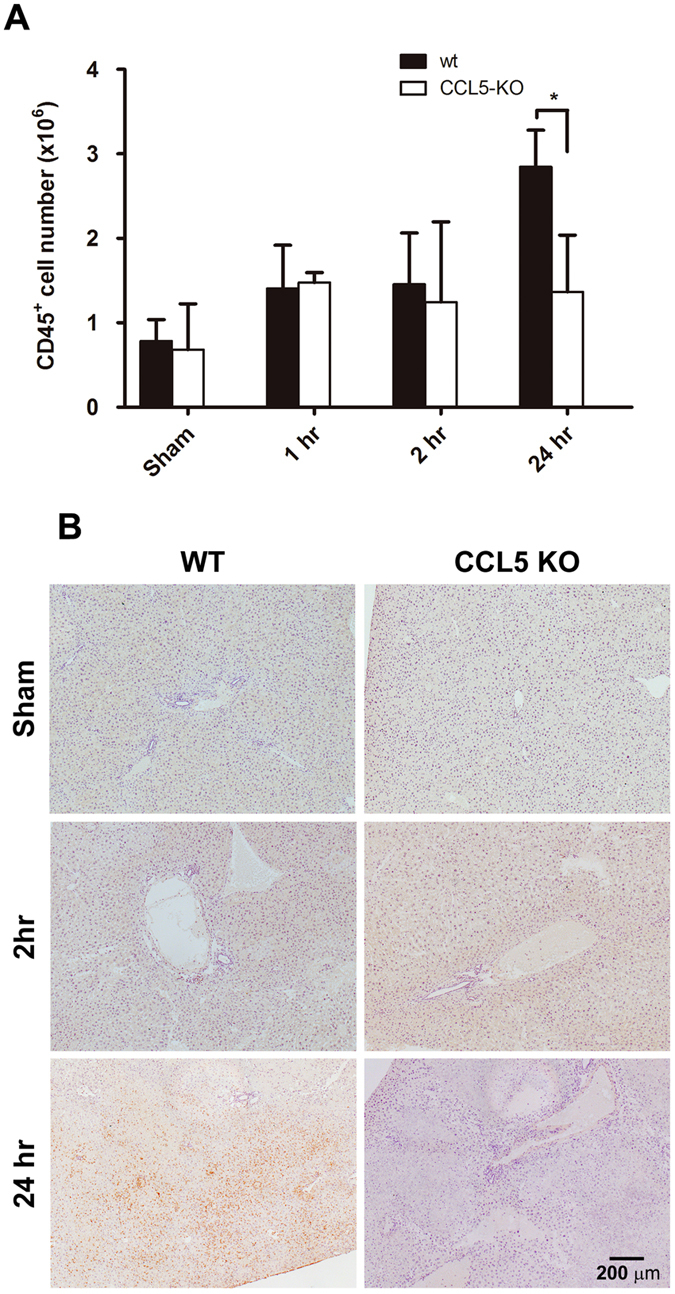
Assessment of intrahepatic leukocyte recruitment by flow cytometry. Intrahepatic leukocytes fractions were isolated from mice subjected to 1 hr ischemia and different reperfusion length (wt, and CCL5-KO mice), and recruiting leukocytes were analyzed by flow cytometry. (**A**) Quantity of CD45+ cells as a function of reperfusion time. Population percentages of CD45+ cells from the results of flow cytometric analysis were plotted against respective lengths of reperfusion. Mean ± SD, n = 4–7. *p < 0.05. (**B**) Immunohistochemistry of CD45+ cells as a function of reperfusion time. Scale bar = 200 μm.

**Figure 7:**
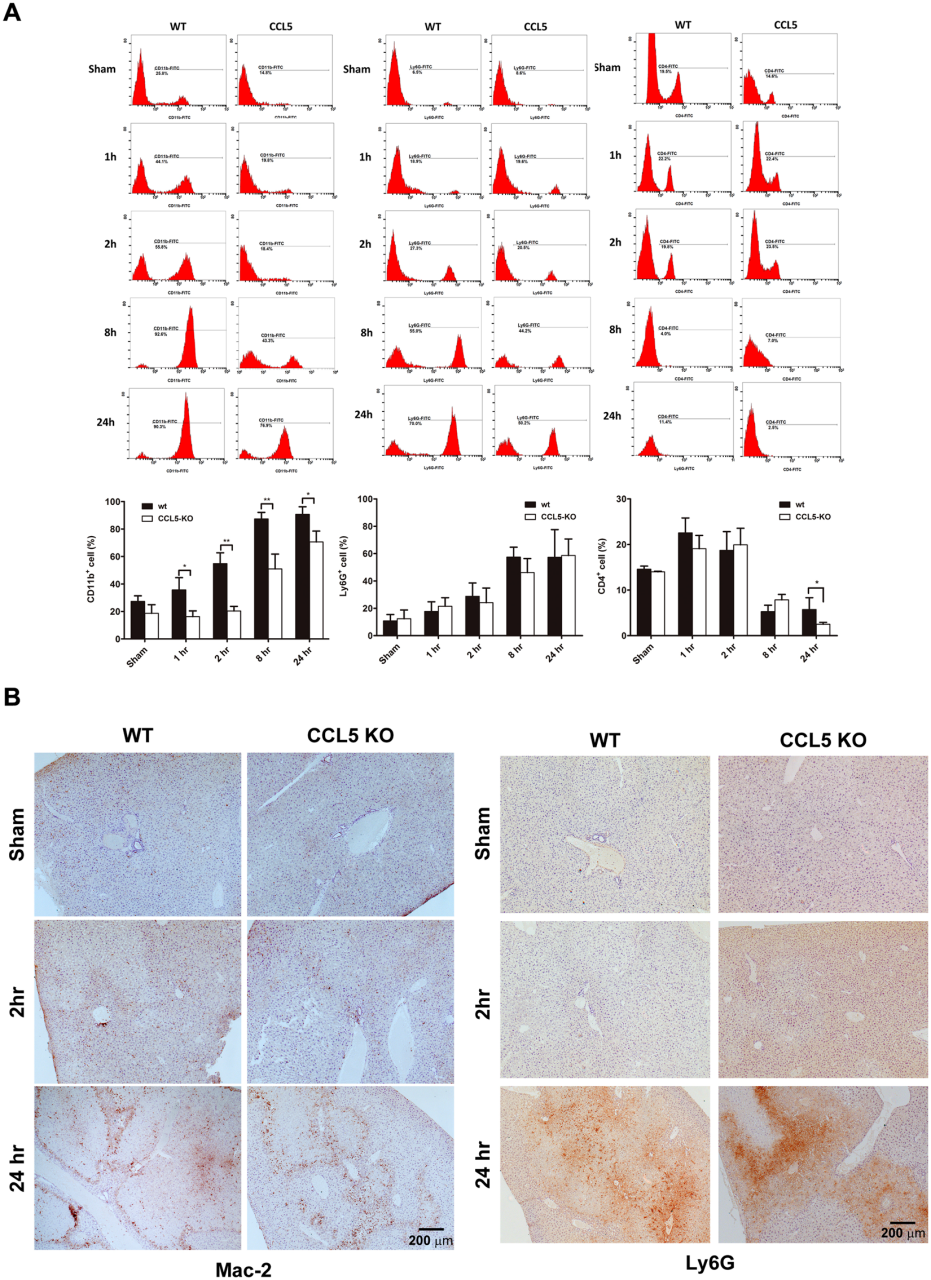
Assessment of intrahepatic leukocyte subsets by flow cytometry. Intrahepatic leukocytes fractions were isolated from mice subjected to 1 hr ischemia and different reperfusion length (wt, and CCL5-KO mice), and recruiting leukocytes were analyzed by flow cytometry. (**A**) The flow cytometry histograms are representative of at least three independent experiments. Numbers indicate the population percentages of CD11b+, Ly6G+, and CD4+ cells in each histogram. Population percentages of CD11b+, Ly6G+, and CD4+ cells from the results of flow cytometric analysis were plotted against respective lengths of reperfusion. Mean ± SD, n = 4–7. *p < 0.05; **p < 0.01. (**B**) Immunohistochemistry of Mac-2+ and Ly6G+ cells as a function of reperfusion time. Scale bar = 200 μm.

To characterize the extent of leukocyte involvement in CCL5-mediated pro-inflammatory response to LIR injury, post-ischemia liver lysates containing infiltrating leukocytes were isolated from wt or CCL5-KO mice at 1, 2, and 24 hrs post-reperfusion. Flow cytometric enumeration of leukocytes (CD45+) by cellular number revealed that wt mice with significantly increased leukocytes as compared to CCL5-KO mice at 24 hrs post-reperfusion (2.847 × 10^6^ ± 0.433 × 10^6^ vs. 1.366 × 10^6^ ± 0.671 × 10^6^, p < 0.05) but did not significantly varied at 1 hr (1.404 × 10^6^ ± 0.514 × 10^6^ vs. 1.477 × 10^6^ ± 0.118 × 10^6^, p > 0.05), and 2 hrs (1.456 × 10^6^ ± 0.607 × 10^6^ vs. 1.246 × 10^6^ ± 0.949 × 10^6^, p > 0.05) post-reperfusion (Fig. 6A). Similar trend was observed in immunohistological staining of the hepatic IRI tissues of CCL5-KO mice using anti-CD45 antibody, which revealed a dramatic reduction of infiltrated CD45^+^ leukocytes (Fig. 6B). We further gated the isolated leukocytes using immunocytometric staining with FITC-conjugated antibodies targeting CD11b, Ly6G, and CD4, which are representative markers of murine myeloid cells (predominantly macrophage), neutrophil, and T lymphocytes, respectively, to identify these three leukocyte subsets. The percentages of CD11b^+^, Ly6G^+^, and CD4+ cells were determined at 1, 2, 8, and 24 hours after reperfusion by flow cytometric assay. Of all three liver-infiltrating lineages, only CD11b+ cells displayed a significant percentage drop in CCL5-KO mice as compared to wt control at liver reperfusion of 1 hr (16.250 ± 4.172 vs. 35.725 ± 8.892, p < 0.05), 2 hrs (20.350 ± 3.323 vs. 54.825 ± 7.862, p < 0.01), 8 hrs (50.950 ± 10.819 vs. 87.300 ± 4.762, p < 0.01), and 24 hrs (70.600 ± 7.920 vs. 90.700 ± 5.459, p < 0.05) (Fig. 7A). A similar result was observed in immunohistochemistry stains of Mac-2 and Ly6G in tissue sections of liver 2 hours and 24 hours post reperfusion after 60-minute ischemia (Fig. 7B).

### CCL5 from hepatic tissue facilitates chemotaxis of circulating macrophages

**Figure 8:**
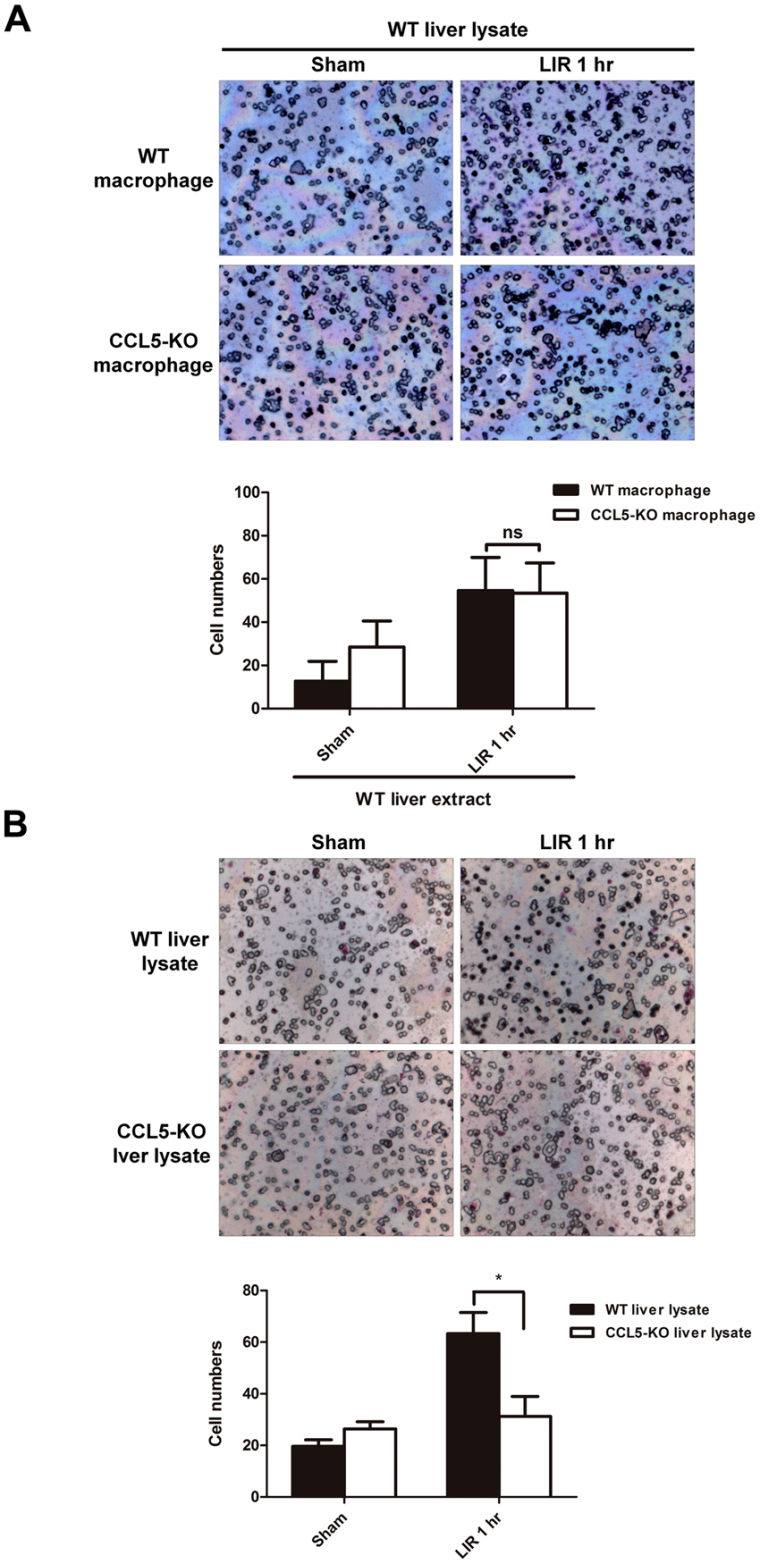
CCL5 signaling is essential of macrophage chemoattraction in liver ischemia/reperfusion. (**A**) Thioglycollate-elicited WT and CCL5-KO mice peritoneal macrophages were harvested, and migratory activity of WT and CCL5-macrophage in response to liver lysate of WT mice for 3 hr. Typical images are representative from three independent experiments. The chemoattractive activity was measured by counting the migratory cellular numbers from 5 random captured field in per individual experiment. Static data (bottom panel) was calculated from three individual experiments. Mean ± SD, n = 3–5. ns = not significant at p > 0.05. (**B**) Thioglycollate-elicated WT mice peritoneal macrophages were harvested, and migratory activity of WT-macrophage in response to liver lysate of WT or CCL5-KO mice for 3 hr. Typical images are representative from three independent experiments. The chemoattractive activity was measured by counting the migratory cellular numbers from five random captured field in per individual experiment. Static data (bottom panel) was calculated from three individual experiments. Mean ± SEM. *p < 0.05.

To characterize whether CCL5 gene deficiency in macrophages affected cell migration ability, we performed a chemotaxis assay with the bottom chamber filled with liver lysate obtained from WT livers that were either ischemia/reperfusion (1 hr/1 hr)-insulted or sham-operated as control. Primary macrophages harvested from WT or CCL5-KO mice were placed in the top chamber and allowed to migrate through the membrane partition for 3 hours. We observed no significant difference between WT and CCL5-KO primary macrophages in the number of migrating cells per assay in response to post-ischemia/reperfusion or sham-treated WT liver lysates [Fig. 8A; “sham”: 28.5 ± 12.021 (CCL5-KO macrophages) vs. 12.667 ± 9.238 (WT macrophages), p > 0.05; “LIR 1 hr”: 53.429 ± 13.939 (CCL5-KO macrophages) vs. 54.600 ± 15.339 (WT macrophages), p > 0.05). We thus further assessed the contribution of CCL5 expression in mice liver as a chemoattractant to circulating macrophages. The liver of WT or CCL5-KO mice was obtained after sham hepatic operation or post hepatic ischemia/reperfusion (1 hr/1 hr) and placed in the bottom chamber as the chemoattractant phase. Primary macrophages from WT animals were then placed in the top chamber to allow migration through the membrane partition for 3 hours. Macrophage migrated to post-ischemia/reperfusion CCL5-KO liver lysate at a significantly fewer number per assay than they did to post-ischemia/reperfusion WT liver lysate (Fig. 8B; “LIR 1 hr”; CCL5 vs WT: 31.20 ± 7.736 vs. 63.33 ± 8.140, p < 0.05). In contrast, there was no appreciable difference in the ability of liver lysates of sham-treated WT or CCL5-KO livers to attract macrophages (Fig. 8B; “Sham”; CCL5 vs WT: 26.40 ± 2.766 vs. 19.67 ± 9.559, p > 0.05). Thus, CCL5 gene expression plays a critical role in macrophage infiltration at early reperfusion stage after liver ischemia (1 hr).

## Discussion

This study investigated the role of Chemokine (C-C motif) ligand 5 (CCL5) as a key mediator of hepatic ischemia–reperfusion injuries (IRI), which are common postoperative hepatic complications. CCL5, a versatile member of the C–C chemokine family, mediates chemotaxis and leukocyte activation through interactions with C–C chemokine receptor type 1 (CCR1), CCR3, and CCR5 and through an oligomerization-dependent interaction with cell surface glycosaminoglycans, respectively^4, 17^. The role of CCL5 in inflammatory disorders and its complex influence on the prevention of HIV entrance and, in some circumstances, the enhancement of HIV replication^4^ have received increased attention. Nevertheless, relatively few studies have investigated the role of CCL5 in hepatic IRI prognosis. Notably, the onset and development of IRI and subsequent aggravation can be considered cascading events of inflammatory responses^18, 19^. From this perspective, the active roles of inflammatory factors have improved our understanding of IRI.

CCL5 is expressed in various cells, including CD8+ T cells, macrophages, platelets, eosinophils, fibroblasts, as well as endothelial, epithelial, and endometrial cells^4, 5^. Our previous studies have demonstrated that CCL5 and interferon-gamma-inducible protein 10 are the most prominently expressed chemokines among those upregulated in murine hepatic and renal IRI models of the C57BL/6C background with congenic A2a adenosine receptor-knockout (A_2A_AR-KO) and A_2A_AR-KO/wt bone marrow transplantation (BMT) chimeras^14, 15, 20^. In the present study, the inhibition of CCL5-mediated signaling was found to significantly ameliorate hepatic IRI in the murine hepatic ischemia/reperfusion (I/R) model. Furthermore, CCL5 exerted positive effects on hepatic IRI aggravation by introducing recombinant mouse (rm)CCL5 in the hepatic I/R model immediately after reperfusion. The introduction of rmCCL5 necessitated the reduction of the ischemia study duration to 30 minutes to avoid the interference of outright mortality while investigating the aggravating effects of rmCCL5. In addition, the functional study of hepatic IRI with deficient CCL5 expression showed alleviated severity of IRI-related inflammation, confirming the crucial role of CCL5 in hepatic IRI development.

We evaluated leukocyte infiltration characterized by CD45 expression and CD11b+ and Ly6G+ cell infiltration represented by murine macrophage and neutrophil populations, respectively. The timing of neutrophil recruitment in an I/R model remains debatable because previous I/R models have reported varying results^21–26^. The infiltration of CD11b+ cells was higher than that of Ly6G+ cells in early postreperfusion. We have used CD11b staining to depict the predominant macrophage population in our previous studies^27, 28^, which is in accordance with published studies^29–32^. In addition to CD11b labeling, marker-based identification is required in order to delineate the specific macrophage population associated with each reperfusion phase. Nonetheless, our results support the notion of possible early infiltration of macrophages or macrophage-like cellular compartments. The present observations are similar with an established model of hepatic IRI-trigerred proinflammatory cascade, in which the release of increased amounts of cytokines or chemokines, such as tumor necrosis factor-alpha (TNF-α), after liver-resident macrophage (Kupffer cells) activation, was possibly one of the major causal factors of late-phase massive neutrophil infiltration^33, 34^. Notably, the importance of early macrophage infiltration in the initiation of proinflammatory injury has been sufficiently reported in several acute inflammatory models, such as renal IRI^35^, myocardial IRI^21^, and acetaminophen-induced liver injury models^36^. The infiltration of circulating monocytes or macrophages, such as CD11b+ cells, during early reperfusion may therefore contribute to further neutrophil recruitment and adhesion because of the secretion of proinflammatory cytokines (e.g., TNF-α and interleukin-1) by such circulating cells as well as resident Kupffer cells. In our previous study, significant neutrophil infiltration started at approximately 4–8 hours after reperfusion^14^, which is consistent with the existing findings on macrophage activation and subsequent neutrophil recruitment^37–39^. Therefore, the available evidence suggests that macrophage infiltration after early reperfusion is possibly concurrent, if not precedent, with early neutrophil infiltration.

Our BMT chimera model showed that CCL5 derived from both circulating BM-derived cells (BMDCs) and parenchymal hepatocytes is required to facilitate the inflammatory aggravation of hepatic IRI because deficient CCL5 expression in either of the two cells resulted in reduced severity of hepatic IRI compared with wt/wt chimera mice. Our findings are consistent with a recent study, which demonstrated that CCL5 inhibition alleviates cardiac and cerebral injuries^6, 13^. In addition, a study on cerebral IRI revealed that blood cell-derived CCL5 causes inflammatory damages^13^. Similarly, Oamada *et al*. explored the role of blood cell-derived CCL5 in promoting inflammation and observed that the production of peripheral blood mononuclear cell-derived CCL5 can be induced in patients with allergic asthma by incubation with a specific mite-allergen^40^. However, CCL5 expression in circulating BMDCs alone was insufficient to maintain the maximum severity of hepatic IRI in an IR event, as shown in the results of our BMT model with a WT BM donor and a CCL5-KO recipient.

The absence of significant differences between the CCL5-KO/WT, WT/CCL5-KO, and CCL5-KO/CCL5-KO groups suggested that (1) tissue-derived CCL5 is necessary but insufficient to trigger IRI-related inflammation and (2) CCL5 expression in circulating BMDCs is one of the two integral components (the other being tissue CCL5 expression) required for IRI development. Therefore, the present results support that both hepatic tissue- and BMDC-derived CCL5 are critical for sustaining the hepatic IRI-triggered inflammatory cascade. These results are in contrast to our expectations because parenchymal hepatocyte- and circulating BMDC-derived CCL5 have been assumed to facilitate proinflammatory events in a synergistic or additive manner. Previous studies have reported that interstitially expressed CCL5 maintains the activity of resident macrophages and protects them against hazardous incident-induced apoptosis^41, 42^. A possible explanation for this dual requirement in the development of hepatic IRI-triggered proinflammatory events may be that tissue-derived CCL5 maintains the inflammatory activity of resident myeloid cells during the early hepatic IRI phase to afford the subsequent recruitment and activation of nonresident cells and further propagation of the proinflammatory cascade. Because the reactive oxygen species and proinflammatory cytokines released by resident macrophages (i.e., Kupffer cells) are crucial for initiating and propagating cellular damage in the early hepatic IRI phase^34^, we suspected that the absence of hepatic tissue CCL5 expression might increase the risk of inflammation-induced injury in hepatic macrophages, thus inducing apoptosis. The heightened elimination of hepatic macrophages post-IRI without CCL5 protection would consequently suppress the paracrine production of proinflammatory cytokines and chemokines, further inhibiting the initiation of the downstream proinflammatory cascade. However, the possibility cannot be neglected that the BMT chimera models may occasionally mask the individual variations and consequently not exhibit net significant differences among the tested donor/recepient groups of CCL5-KO/WT, WT/CCL5-KO, and CCL5-KO/CCL5-KO. Furthermore, an unknown lineage recruitment and interaction pathway may be present, and this may release a substantial amount of CCL5 to participate in IRI-induced inflammation. Therefore, further investigation is warranted to explore such possibilities.

Our liver lysate chemotaxis assay results showed that hepatic tissue CCL5 expression is required for BMDC (isolated circulating macrophages) recruitment. Regarding the dual requirement of CCL5 expression in hepatic tissue and circulating BMDCs for complete hepatic IRI development, CCL5-mediated cell recruitment in hepatic tissues indicates a critical role of tissue CCL5 in facilitating the recruitment of circulating macrophages during early hepatic IRI phase (at 1-hour postreperfusion in our study). Although our results do not preclude CCL5-mediated circulating cell recruitment or cell–cell recruitment and interaction in the liver parenchyma, such recruitment most likely occurs as late events after the initial recruitment and activation of these cells to the injured hepatic tissues, possibly through sinusoid endothelium deregulation.

Altogether, our results suggest that hepatic tissue CCL5 expression is responsible for the initial sustainence of resident macrophages in response to hepatic IRI-induced inflammation and the recruitment of circulating BMDCs. In addition, CCL5 expression in circulating BDMCs is required for complete post-IRI inflammatory cascade represented by massive cellular infiltration and the proliferation of proinflammatory signals at 24 hours postreperfusion and beyond, thus leading to the conflagrating amplification of post-IRI inflammation^43, 44^. Therefore, tissue CCL5-facilitated hepatic infiltration of circulating macrophages during the early hepatic IRI phase is critical to subsequent proinflammatory damages. This early recruitment consequently triggers the massive recruitment of neutrophils, probably through the continual recruitment and activation of circulating macrophages, which release cytokines or chemokines upon activation. CCL5 acts directly on macrophages but not on neutrophils during hepatic IRI; this finding is consistent with both a previous finding that the functional blocking of CCL5 does not exert significant effects on neutrophil chemotaxis^45^ and the present finding that CCL5 KO does not significantly affect neutrophil infiltration during hepatic IRI.

In conclusion, our data demonstrate the pathway of CCL5-mediated hepatic IRI progression, in which the development of proinflammatory events above the baseline responses requires CCL5 expression in the hepatic tissue and myeloid components. Furthermore, the phenomenon confirms the pleiotropic nature of CCL5 during sterile inflammation as being similar to that observed in hepatic IRI.

## Methods

### Animals

C57BL/6C mice were purchased from Charles River Taiwan Branch (male, 10–12 wk of age, BIOLASCO LTD, Taiwan). The congenic CCL5-KO mice (male, 10–12 wk of age) in C57BL/6 C background used in our studies were originally obtained from the Jackson Laboratory and backcrossed at least 20 generations with C57BL/6 C in Chang Gung Memorial Hospital (CGMH). All animals were housed within a barrier facility in the Laboratory Animal Center of CGMH. All procedures of husbandry and animal experiments followed the guidelines of the National Institutes of Health and were approved by the Institutional Animal Care and Use Committee of CGMH. Mice were bred and maintained in a 12-hour light/12-hour dark cycle at a temperature of 20 ± 3 °C with relative humidity at approximately 55%. Food and water were provided ad libitum.

### Liver IRI and drug treatment

Mouse model of partial warm hepatic IRI was produced as described by our earlier report^15^. In brief, a midline laparotomy was made to expose the porta hepatis. Partial hepatic ischemia was induced by applied a microaneurysm clip to the hepatic triad above the bifurcation in order to clamp the blood flow of the left hepatic artery, portal vein, and bile duct. The right hepatic artery and right lobe remained intact. For most of the studies, the microaneurysm clip was removed after 60 minutes ischemia. For studies administrated with recombinant mouse CCL5, the clip ischemia time was 30 minutes. Immediately before reperfusion was initiated, each mouse received either of the following treatments: a single bolus i.v. injection of anti-CCL5 Abs (1 ng, 10 ng, 100 ng, ACRIS PP1081P1, NY USA); control IgG (100 ng, ACRIS AP22127PU-N, NY USA); Met-CCL5 (10 ng, 100 ng, 1,000 ng, R&D 335-RM/CF, USA); or recombinant mouse CCL5 (1 ng, 10 ng, 100 ng, PeproTech 250-07, USA). After the 24 hr reperfusion, animals were reanesthetized, blood was obtained by cardiac puncture, and liver was removed for various analyses. A sham control animal that underwent a midline laparotomy and closure without hepatic vascular clamping was also included in this study.

### Bone marrow transplantation (BMT)

The details of the BMT procedures were described by us^15^. In brief, wt or CCL5-KO C57BL/6 C mice at age of five weeks were irradiated and used as recipients for BMT experiments. Donor marrows were derived from either one of the C57BL/6 C, wild type (WT), or congenic knockout mice of CCL5 in B6 background. BM cells were isolated form male donor mice (12 wk old; 25–28 g), and 3 × 10^6^ BM cells were injected intravenously to recipients of WT or CCL5-KO. Irradiated/transplanted mice were housed in isolation room with IVC for at least 8 weeks before experimentation and given autoclaved food and acid water containing 5 mM sulfamethoxazole and 0.86 mM trimethoprim.

### Serum alanine aminotransferase (ALT) activity

Whole blood was obtained by cardiac puncture and placed on ice before centrifugation at 14,000 × g for 20 min. Serum was collected and stored at −80 °C. Alanine Aminotransferase (ALT) was measured by VTROS DT60 II Chemistry System. In brief, the frozen serum samples were thawed and diluted 5–10 times with pre-warm saline. Each of 10 μl serum was added to VITROS ALT DT Slide by manufacture’s pipette. The measuring process was an automatic operation carried out by the VTROS DT60 II Chemistry System. The levels of ALT were expressed in International Unit. The results were collected and calculated with Prism 5.0 (GraphPad, USA).

### Myeloperoxidase (MPO) assay

The MPO assay was performed as described in our previous publication^14^. In brief, mouse livers were removed after liver IRI. The liver was homogenized, centrifuged, and the supernatant was discarded. The pellet was washed, resuspended, and sonicated, and then subjected to three freeze/thaw cycles. After centrifugation, supernatant was harvested and added to an equal volume of a solution consisting of *o*-dianisidine (10 mg/ml), 0.3% H_2_O_2_, and 50 mM KPO_4_, pH 6.0. Absorbance was measured at 460 nm over a period of 5 min.

### Histology

Livers were harvested after indicated time of reperfusion, fixed in 4% paraformaldehyde in PBS (pH 7.4), and embedded in paraffin. Four-micrometer sections were subjected to standard H&E staining and viewed by light microscopy (Zeiss AxioSkop). The resulting images were captured under a microscope (AxioplanII; Carl Zeiss Microimaging Inc., Germany), and brightness/contrast adjustment was made with AxioVision software.

### Immunohistochemistry studies

For immunohistochemistry studies, 4-μm tissue sections were incubated with primary antibody followed by a biotinylated secondary antibody according to the manufacturer’s protocol of Vectastain Elite ABC Kit (Vector Labs, Burlingame, CA, USA). The primary antibodies were used in these experiments were as follows: Rat anti-MAC2, Rat anti-Ly6G, and Rat anti-CD45 (BD Biosciences Pharmingen, San Diego, CA, USA).

### Cell preparation and Flow cytometry

The median and left lobes of hepatic tissue were harvested from mice subjected to 1 hr ischemia followed by reperfusion for indicated time. The freshly harvested lobes were minced in RPMI1640 and ground to pass through a gauze swab and then filtered by a 70-μm nylon mesh. The leukocytes fractions were isolated first by 35% Percoll density grandient, followed by RBC lysis with ACK lysing buffer (0.15 M NH_4_Cl, 1 mM KHCO_3_, and 0.1 mM Na_2_-EDTA, pH 7.4). The final cell pellet was washed with ice-cold 1 × PBS and resuspended in flow cytometry staining buffer (eBioscience) following the manufacture’s protocol. In brief, 0.1-ml of each aliquot was placed on ice and stained for 45 min in the dark with FITC-conjugated specific antibodies respectively against mouse CD45, CD11b, Ly6G, and CD4 (eBioscience, San Diego, CA, USA). Stained cells were washed and resuspended with iced PBS. The fluorescence intensity was measured with excitation wavelength of 488 mm and emission wavelength of 530 mm and a minimum of 40,000 events being collected, data were acquired and analyzed using flow cytometry (Cytomics FC500; Beckman Coulter, Inc, Fullerton, CA). The total CD45+ cell numbers were calculated as follows: [The final harvest of total cell numbers by 35% Percoll density grandient and RBC lysis] × [the ratio of CD45+ cell population in total cells as detetermined by flow cytometry].

### Isolation peritoneal macrophages and transwell migration assay

Macrophages were elicited by i.p. injections of 1 mL 3% Brewer thioglycollate broth (Sigma) in B6 mice receiving by 2 duration interval day. After the 8th day, the macrophages were collected by peritoneal lavage with 5 ml RPMI (with 1 mM EDTA). To purify the macrophages, the peritoneal lavage were centrifuged (2,000 × g for 5 min), washed, and suspended with serum-free RPMI medium. Single cell suspension further plated into Petri dishes for 4 hr; the nonadherent cells were removed by PBS wash, and then the adherent cells were subjected to flowcytometric assay. More than 99% of attached primary macrophages were positive for CD11b and F4/80, were used in these migration assays. Attached macrophages were overnight cultured in RPMI medium (with 10% FBS), and cells were lifted by PBS with 10 mM EDTA and cell scrapers after 2 hrs serum starvation. 2 × 10^5^ dispersed cells were resuspended into RPMI medium (with 1% FBS) and seeded into 24-transwell inserts with 8 μm diameter pore polycarbonate membrane (Millipore Corp.) for 3-hr migration assay in the lower chambers were filled with 1 mL of liver lysate from mice with insult of liver ischemia (1 hr)/reperfusion(1 hr). The liver lysate was generated as follows: Left liver lobe was aseptically excised, rinsed with sterilized ice-cold PBS containing 200 U/mL Penicillin-Streptomycin (Invitrogen), and then minced into small pieces by 100 μm nylon mesh, and subsequently resuspended in 10 mL of DMEM (1% FBS) medium, and passed through a 40 μm filters followed by centrifugation of 800 g × 3 min at 4 °C, and discard tissue derbies to harvest liver lysate.

### Statistical analysis

The graphic and statistical analyses were performed using the scientific graphing software Prism 5.0 (GraphPad, USA). Unpaired Student’s t test was used for all comparisons in this study. A value of *p* ≤ 0.05 was used to define statistical significance.
